# A British Society of Gastrointestinal and Abdominal Radiology multi-centre audit of imaging investigations in inflammatory bowel disease

**DOI:** 10.1093/bjr/tqaf050

**Published:** 2025-03-10

**Authors:** Katherine Taylor, Elizabeth Robinson, Ravivarma Balasubramaniam, Gauraang Bhatnagar, Stuart A Taylor, Damian Tolan, Anita Wale, Ian Zealley, Kieran G Foley

**Affiliations:** Department of Radiology, National Imaging Academy of Wales, Pencoed, CF35 5HY, United Kingdom; Department of Radiology, Royal United Hospitals Bath NHS Foundation Trust, Bath, BA1 3NG, United Kingdom; Department of Radiology, Royal Stoke University Hospital, ST4 6QG, United Kingdom; Department of Radiology, Frimley Health NHS Trust, Frimley, GU16 7UJ, United Kingdom; Centre for Medical Imaging, University College London, London, WC1E 6BT, United Kingdom; Centre for Medical Imaging, University College London, London, WC1E 6BT, United Kingdom; Department of Radiology, St James’s University Hospital, Leeds Teaching Hospitals NHS Trust, Leeds, LS9 7TF, United Kingdom; Department of Radiology, St Georges NHS Foundation Trust & St Georges University of London, SW17 0QT, United Kingdom; Department of Radiology, Ninewells Hospital, NHS Tayside, Dundee, DD2 1SG, United Kingdom; Division of Cancer and Genetics, School of Medicine, Cardiff University, Cardiff, CF14 4XN, United Kingdom

**Keywords:** inflammatory bowel disease, small bowel, imaging, enterography, MRI, ultrasound, CT, radiography

## Abstract

**Objectives:**

To evaluate current UK practice for inflammatory bowel disease (IBD) imaging against recommendations from published international literature.

**Methods:**

A retrospective multi-centre audit was undertaken evaluating imaging modalities, protocols, and pathways used to investigate IBD both in outpatient and inpatient settings during January-December 2022. Reporting practices and training provisions were also recorded.

**Results:**

Forty-one centres contributed: 35 centres provided complete data, whereas 6 centres provided incomplete data. Magnetic resonance enterography (MRE) was the most common modality for small bowel imaging across UK centres, comprising 13 099/18 784 (69.7%) investigations. There was regional variability in other modalities used, with 5 centres performing 81% of all intestinal ultrasound and 3 centres performing 65% of all small bowel follow-through. Compared with outpatients, inpatients with suspected IBD were significantly more likely to be imaged with techniques imparting ionising radiation whether scanned either in-hours (*p* = 0.005) or out-of-hours (*p* < 0.001). Non-ionising radiation imaging modalities were significantly less available out-of-hours (*p* < 0.0001). Sequences included in MRE protocols were variable. Disparity in imaging follow-up for patients prescribed biologic therapies was observed.

**Conclusions:**

Considerable variation in UK IBD imaging practice has been identified. Improvements must be made to reduce the regional inequality of patient access to different imaging modalities and decrease reliance on ionising radiation for inpatients. Further research to standardise and optimise imaging pathways should be undertaken to improve uniformity, with emphasis placed on training and education.

**Advances in knowledge:**

This multi-centre audit showed considerable IBD imaging practice variation between UK centres, particularly for imaging modalities used between inpatient and outpatient groups and in-hours versus out-of-hours.

## Introduction

Inflammatory bowel disease (IBD) is characterised by acute and chronic inflammation of the gastrointestinal tract and consists of 2 main entities, Crohn’s disease and ulcerative colitis.^1^^,^^2^ Inflammatory bowel disease affects over 500 000 people in the United Kingdom (UK) and is commonly diagnosed between the ages of 15 and 40.^3^ It carries significant morbidity for those affected, often requiring repeated investigations for disease flares and monitoring therapeutic response.^4^

The diagnosis and management of IBD are complex and mandate an interdisciplinary approach.^2^^,^^5^ Radiological imaging is seen as complementary to endoscopic assessment, playing a crucial role in assessing the extent, phenotype, activity, and severity of small bowel disease proximal to the terminal ileum that cannot be accessed with conventional ileocolonoscopy, whilst also evaluating for complications, such as penetrating or stricturing disease. Inflammatory bowel disease management has moved away from symptom control towards earlier, more aggressive, combined immunosuppression to achieve remission. Imaging plays a pivotal role in this treat-to-target strategy.

In 2011, a survey of UK imaging practice identified small bowel follow-through (SBFT) as the predominant imaging modality used for investigating suspected or known small bowel Crohn’s disease, with magnetic resonance enterography (MRE) and intestinal ultrasound (IUS) used less frequently.^6^ However, current UK and European guidelines now discourage the routine use of barium studies and place emphasis on non-ionising imaging modalities, particularly given the young patient demographic and frequent need for repeated examinations.^4^^,^^7^ These guidelines also highlight the importance of early diagnosis and accessibility to cross-sectional imaging within 24 hours in patients who are acutely unwell or within 4 weeks for non-acute patients.^4^ Delays in diagnosis and treatment of IBD are associated with adverse outcomes, including increased risk of disease progression, complications, and higher rates of emergency surgery.^8^

The increased demand for imaging, variation in access to non-ionising imaging out-of-hours, and workforce challenges place considerable pressure on radiology departments and can impact the delivery of optimal patient care for multiple conditions, including IBD. The primary aim of this audit was to evaluate current imaging practice for IBD in the NHS against published UK and European guidelines.

## Methods

A retrospective, national, multi-centre audit was conducted by the British Society of Gastrointestinal and Abdominal Radiology (BSGAR). An open invitation was sent to BSGAR members working at National Health Service (NHS) Trusts across the United Kingdom to complete an audit questionnaire evaluating practices at their local trust. Recommendations in published literature from IBD UK, National Institute for Health and Care Excellence (NICE), British Society of Gastroenterology (BSG), European Society of Gastrointestinal and Abdominal Radiology/European Society of Paediatric Radiology (ESGAR/ESPR), and European Crohn’s and Colitis Organisation (ECCO)-ESGAR were used to generate the audit standards (Supplementary Material).^4^^,^^5^^,^^7^^,^^9–13^ This audit was conducted within clinical governance guidance and did not record any patient identifiable data; therefore, no formal research ethics committee approval was required.

The first section of the questionnaire focussed on IBD services and imaging techniques. Modalities used for small bowel imaging at each centre and the total number of investigations performed in one calendar year (January 1, 2022 to December 31, 2022, inclusive) were recorded. The techniques and protocols for each imaging modality, parameters measured for IBD assessment, structured reporting use, and training were also included (Supplementary Material).

The second section focussed on IBD imaging pathways in different patient groups: patients with suspected but undiagnosed IBD, confirmed IBD, and treatment response monitoring. This encompassed both elective and acute work in and out-of-hours. For inpatients, normal working hours were defined as 08:00 am to 5:00 pm Monday to Friday, and out-of-hours as 5:00 pm to 08:00 am weekdays or anytime at weekends or bank holidays. Centres were asked to record all imaging modalities routinely used in each setting; the exact number of investigations performed in each setting was not recorded. Centres reported the maximum waiting time for routine outpatient examinations for each modality.

Participants submitted data using the REDCap (Research Data Collection Service) secure database in methods described previously.^14^^,^^15^ Where necessary, duplicate data entries were compared and discrepancies were clarified with individual centres before analysis. Data were then exported to Microsoft Excel 365 for statistical analysis. In each audit section, centres that provided incomplete or missing data were excluded from analysis for each relevant section. Data were summarised using descriptive statistics. Fisher exact test was performed to assess for statistically significant associations between categorical variables. A *p*-value of <0.05 was considered statistically significant.

## Results

Forty-one centres (24 secondary and 17 tertiary centres) from across the UK provided data for the audit. Three centres provided duplicate data. Thirty-five centres completed the questionnaire in its entirety, with 6 centres providing data for some, but not all, sections of the questionnaire.

### Imaging modalities

**Figure 1.**
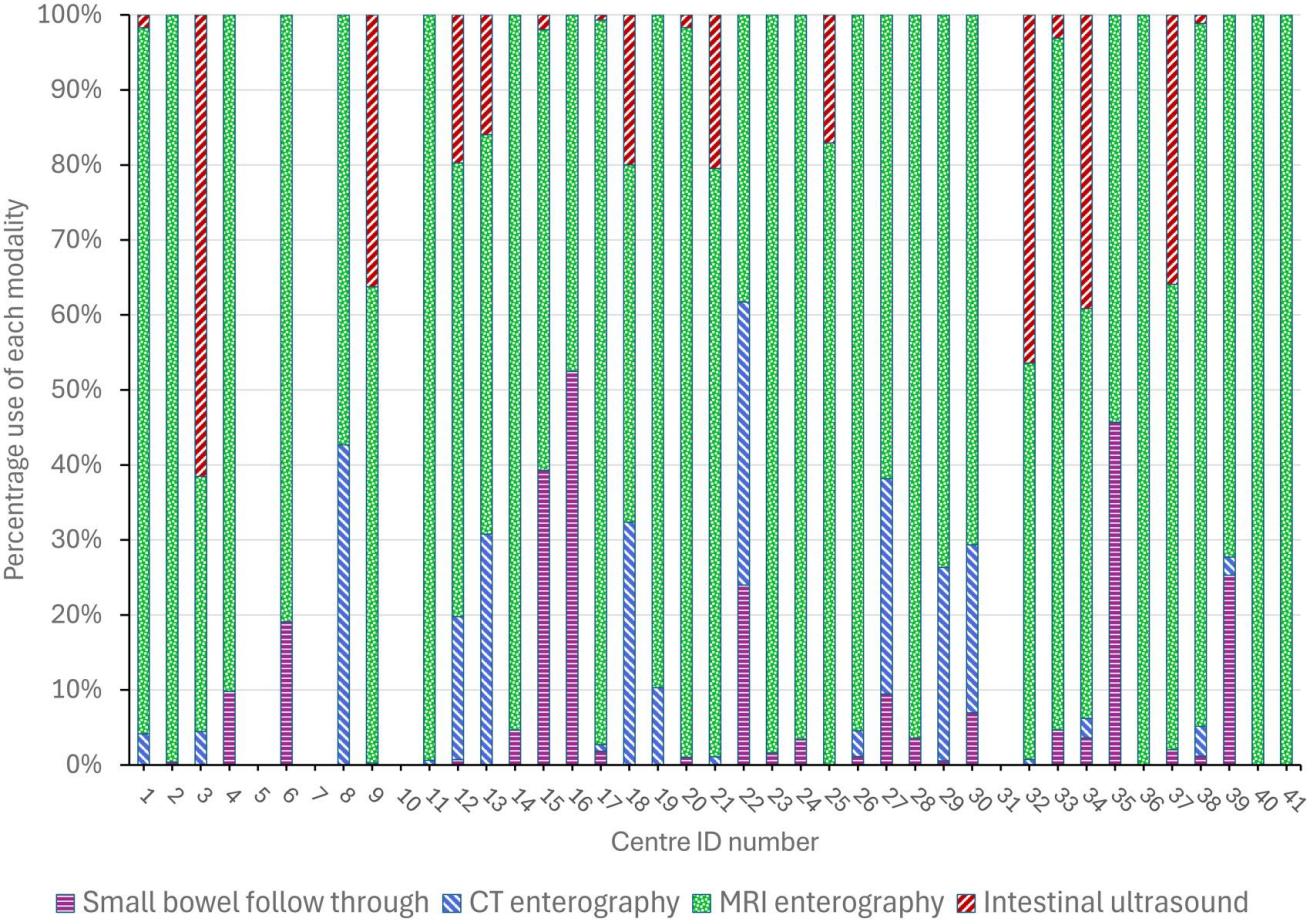
Reported percentages of each imaging modality performed for investigation of the small bowel at each centre in one calendar year.

Forty of 41 (98%) centres used MRE for investigating the small bowel, with computed tomography enterography (CTE) used by (28/41 [68%]), SBFT used by (22/41 [54%]), and IUS by (19/41 [46%]). Thirty-seven of the 41 centres (90%) provided information on the total number of each investigation performed in one calendar year for all IBD indications. Of the 18 784 examinations performed across all centres, 16 365 (87%) used non-ionising modalities (Table 1). Overall, MRE had the highest use, making up >50% of examinations at 33/37 (89%) centres and ≥90% of examinations in 16/37 (43%) centres. However, considerable regional variability in the use of each modality was observed (Figure 1). Although 46% of centres reported using IUS, 5 centres performed 81% of all IUS examinations. On average, these 5 centres used IUS for 44% of their total examinations. Six centres performed 10 or fewer IUS examinations annually. Three centres were high-volume users of SBFT, performing 65% of all SBFT across UK centres, with SBFT comprising an average of 46% of their total examinations. Two of these centres did not provide an IUS service, with the third centre using IUS for 2% of their total examinations. These three centres supplemented SBFT with MRE, which comprised 54% of their total examinations.

**Table 1.** Number of centres that reported using each modality for imaging the small bowel in inflammatory bowel disease (*n* = 41).

Modality	Centres using modality (*n* = 41)	Total number of examinations in 1 year across 37 centres	Mean number of examinations	Range in number of examinations	Percentage of total examinations across 37 centres
Small bowel follow-through	22 (54%)	1027	28	1-371	5.50
CT enterography	28 (68%)	1392	38	2-324	7.40
MRI enterography	40 (98%)	13 099	354	60-1746	69.70
Intestinal ultrasound	19 (46%)	3266	88	3-997	17.50

Of the 41 centres, 37 provided information regarding total number of examinations performed in 1 year.

### Imaging techniques

Data regarding patient preparation for MRE, IUS, and CTE examinations can be found in the Supplementary Material.

#### MRI enterography

##### Sequences

Thirty-seven centres provided data on MRE sequences. Fast spin echo non-fat-saturated T2-weighted (T2w) sequences are recommended by ESGAR/ESPR in both the axial and coronal planes^12^; both were performed in 28/37 (76%) centres, axial only in 4/37 (11%), coronal only in 2/37 (5%), and neither at 3/37 (8%, although a fat-saturated sequence was used instead). The recommended axial and coronal steady state free precession gradient echo (SSFP GE) sequences without fat saturation were both performed at 19/37 centres (51%), axial only in 2/37 (5%), coronal only in 6/37 (16%), and neither in 10/37 (27%). An unenhanced coronal T1-weighted sequence (T1w) with fat saturation was used in 31/37 (84%). A fat-saturated T2w sequence is recommended either in the axial or coronal plane; both were performed at 14/37 (38%) centres, axial only in 2/37 (5%), coronal only in 18/37 (49%), and neither in 3/37 (8%) centres.

**Figure 2.**
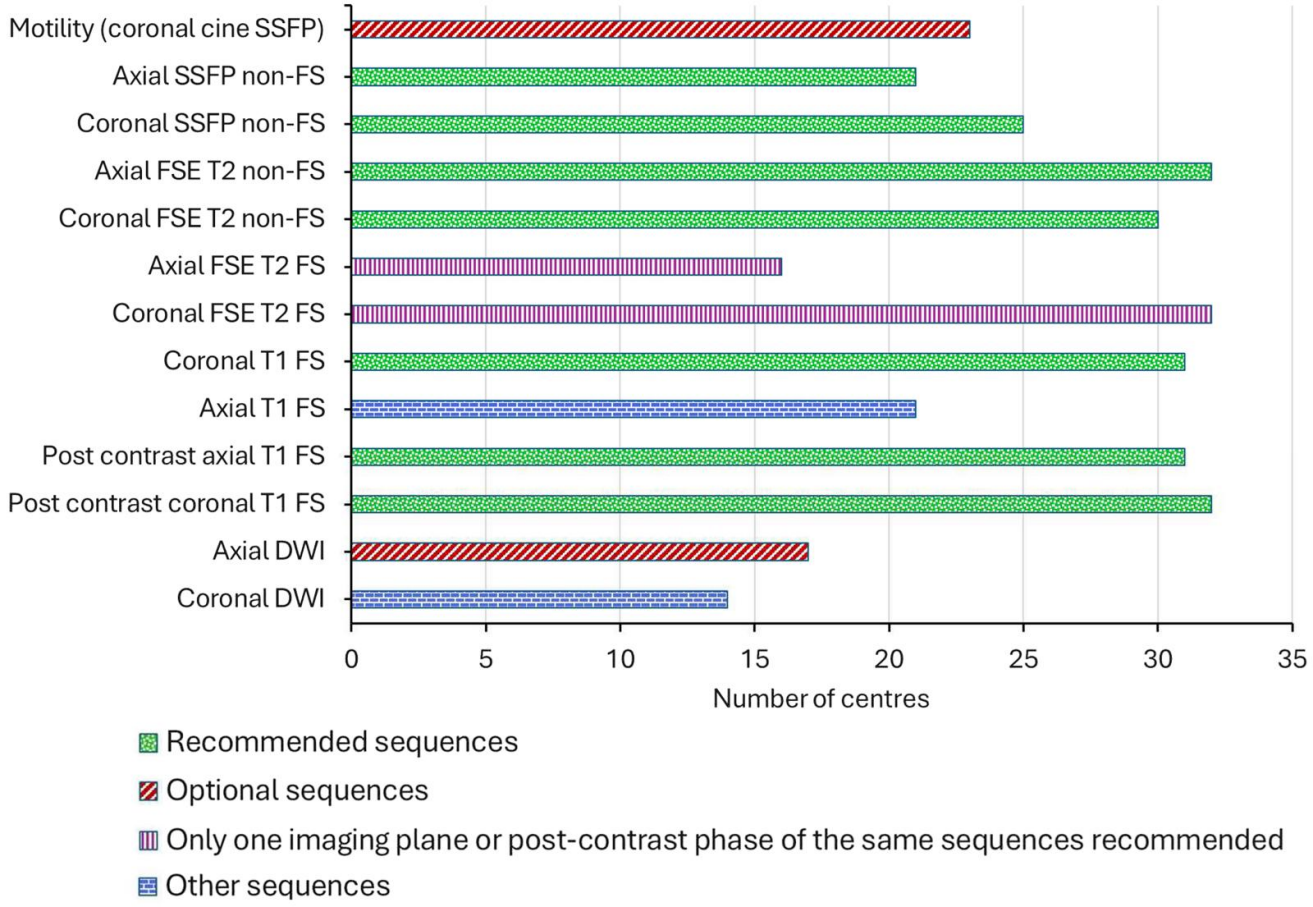
Breakdown of magnetic resonance enterography sequences routinely acquired across 37 centres.

Diffusion-weighted imaging (DWI) use is optional; however, DWI in the coronal plane is not recommended. Although 27/37 (73%) centres used DWI, the imaging plane and *b*-values used were variable. In the 27 centres that used DWI, both axial and coronal DWI were acquired in 4/27 (15%) centres, axial plane only in 13/27 (48%) centres, and coronal only in 10/27 (37%). The recommended low (0-50) and high (600-900) *b*-values were both used at 24/37 (89%) centres. An optional coronal cine motility sequence was performed at 23/37 (62%) centres. Local protocols vary significantly in their inclusion of additional sequences (Figure 2); overall, 4/37 (11%) centres did not use any additional sequences. The mean number of sequences performed was 10 (range 5-15).

Overall, 1/37 centres (3%) provided no information regarding scan time and 2 centres had 2 different scan protocols for different indications; therefore, of the 38 protocols evaluated, 14/38 (37%) performed examinations in under 30 minutes.

##### Intravenous gadolinium

**Figure 3.**
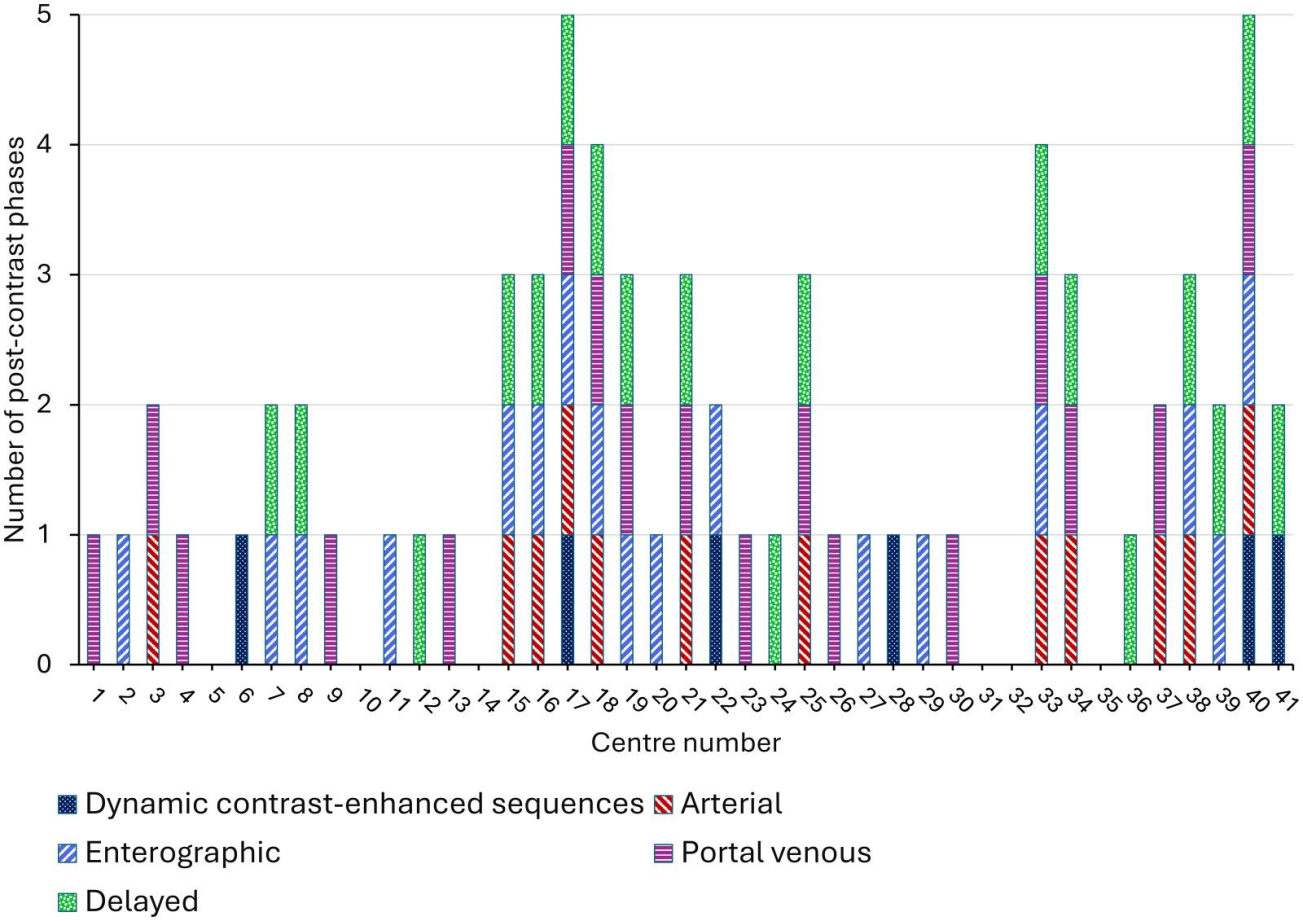
Breakdown of post-contrast phases used across the 35 centres that provided data.

Post-contrast axial and coronal fat-saturated T1w were both routinely used at 30/37 (81%) centres, coronal only at 2/37 (5%) centres, and axial only at one centre (3%). Two additional centres that routinely use a non-contrast protocol for both IBD first assessment and routine follow-up responded to further questions regarding the post-contrast phases used in the event of contrast administration. Therefore, of the 35 centres providing data, there was considerable variation in the contrast phases used, with a median number of phases of 1 and range of 1-5 (Figure 3). Post-contrast imaging is recommended in either the enteric (45 seconds) or portal venous phase (70 seconds)^12^; of the 35 centres, 7/35 (20%) used the portal venous phase only and 5/35 (14%) used the enteric phase only. Ten centres (29%) used one of the recommended phases in combination with other non-recommended post-contrast phases, and 5/35 (14%) centres used both phases, although also combined with other non-recommended phases. The remaining responding centres used non-recommended post-contrast phases. Dynamic contrast-enhanced sequences, which provide a semi-quantitative assessment of perfusion, are not currently recommended but were performed at 6/37 (17%) centres.^16^

##### Non-contrast protocols

In 25/37 (68%) centres, a dedicated non-contrast protocol was available, mostly used in pregnant patients and when intravenous (IV) contrast is contra-indicated (Table 2). Bruining et al^5^ recommend DWI and T2w sequences as acceptable alternatives when IV contrast cannot be administered. Diffusion-weighted imaging was included in the standard non-contrast MRE protocol in 19/25 centres (36%). A non-fat-saturated T2w sequence was included in the standard non-contrast MRE protocol in 24/25 (96%) and a fat-saturated T2w sequence in 22/25 (88%).

**Table 2.** Indications for non-contrast magnetic resonance enterography protocols.

Indication for non-contrast MRE	Number of centres (*n* = 37)
When intravenous contrast is contraindicated	17 (46%)
Imaging in pregnant patients	11 (30%)
Routine IBD follow-up	9 (24%)
Assessment of non-specific abdominal symptoms	8 (22%)
Routine first IBD assessment	8 (22%)
Paediatric protocol	5 (14%)
Standard IBD protocol	1 (3%)

Fisher exact test demonstrated a statistically significant association between the presence of an established imaging pathway for known IBD and the use of non-contrast MRI protocols for routine IBD follow-up (*p* = 0.007). However, there was no significant relationship between the presence of a known IBD imaging pathway and the use of a non-contrast MRE protocol for routine first IBD assessment (*p* = 0.17) or when contrast is contraindicated (*p* = 0.72).

##### MRE imaging parameters and structured reporting

Thirty-nine centres provided information regarding radiologists reporting MRE in their department. In total, 223 radiologists were reporting MRE across the 39 centres, with a median of 5 per centre and a range of 1-19. Data regarding reporting numbers were provided for 181/223 radiologists (81%). Of the 181 radiologists, 156 (86%) had reported >100 cases independently in their careers, 16 reported 50-100, and 12 had reported <50 cases.

Of 37 centres providing data on the parameters used in interpreting MRE imaging, 3/37 (8%) implemented a reporting template (2 secondary care centres and 1 tertiary centre). Quantitative reporting parameters were used variably throughout different centres, with abnormal bowel length and wall thickness measurement being the most frequently reported (Table 3). All centres that used a reporting template recorded abnormal bowel length and wall thickness; one centre included a small bowel motility score.

**Table 3.** Reporting of quantitative magnetic resonance enterography parameters across 37 centres.

Quantitative MRE parameters	Number of centres (*n* = 37)
Abnormal bowel length	32 (86%)
Wall thickness	26 (70%)
Small bowel motility score	3 (8%)
Dynamic contrast enhancement metrics	1 (3%)
None of above	3 (8%)

##### CT enterography

Twenty-one centres (51%) provided completed data on CT scanner capabilities, all of which reported using ≥64 slice multi-detector CT scanner. The maximal slice reconstruction thickness was ≤3 mm standard in 20/21 (95%) centres. Hyoscine butylbromide spasmolytic was administered in 15/21 (71%) centres, and this is listed as optional in the ESGAR/ESPR guidelines. The recommended enterographic (40-60 seconds) or portal venous phase (60-80 seconds) post-contrast phases were used in 20/21 (95%) centres, with the remaining 1 centre performing split-bolus arterial and venous phase imaging. However, only 12/21 (57%) centres calculated administered contrast dose by patient weight.

#### Intestinal ultrasound

##### Technical factors

Seventeen centres completed the questionnaire section regarding IUS protocols. Both a low (2.5-7.5 MHz) and high (>7.5 MHz) frequency probe was used at 14/17 (82%) centres. The remaining 3/17 (18%) centres used high-frequency probes only. The bowel was routinely assessed with colour Doppler in 12/17 (71%). Routine assessment of extra-intestinal organs was performed at 7/17 (41%) centres.

##### Ultrasound training

Of the 19 centres using IUS, 15 completed the questionnaire section regarding IUS training. In 12/15 (80%) centres all radiologists who performed IUS had undergone formal training, with the other 3/15 reporting some but not all radiologists had formal training. Free text responses specifying the types of training received demonstrated a wide variety of responses, including experience during registrar years or fellowships, in-house training, dedicated IUS courses and training during a clinical trial. The exact number of radiologists at each centre who had received each type of training was not recorded.

#### Access to imaging

There is considerable variability in waiting times for outpatient imaging between modalities and centres (Table 4). Mean maximum waiting times are above the IBD UK 4-week target for all modalities,^4^ except for IUS. Magnetic resonance enterography is the most frequently performed modality for general IBD imaging across all centres but had the fewest centres with a waiting time within 4 weeks (11/29 [38%]).

**Table 4.** Reported maximum waiting times for modalities used for imaging inflammatory bowel disease.

Modality	Number of the centres providing data (*n* = 41)	Mean maximum wait time (weeks)	Median maximum wait time (weeks)	Range (weeks)	Centres within 4-week IBD UK target
SBFT	11 (27%)	6.8	3	1-44	9/11 (82%)
MRE	29 (71%)	6.3	6	2-28	11/29 (38%)
CTE	17 (41%)	4.6	4	0-22	11/17 (65%)
IUS	11 (27%)	3.6	2.7	1-8.7	6/11 (55%)

### Imaging pathways

Thirty-five centres provided data regarding IBD imaging pathways. A formal imaging pathway for investigating suspected IBD was present at 11/35 (31%) centres and 16/35 (46%) for known IBD. No statistically significant association between presence of a formal imaging pathway in tertiary versus secondary care centres was demonstrated for suspected IBD (*p* = 0.14) or known IBD (*p* = 0.13).

In the outpatient setting, the median number of modalities employed to investigate suspected, but undiagnosed, IBD was 2 (range 1-4) for those with a suspected IBD pathway, and 2 (range 1-3) for those without a pathway. In inpatients, there was a greater median number of modalities used to investigate suspected IBD in centres without a pathway for suspected IBD (median 3, range 1-4) versus those with a pathway (median 1, range 1-5). Of 6/11 (55%) centres with an imaging pathway for suspected IBD that only used one modality, one centre used IUS alone, with the remaining 5 centres using CT abdomen and pelvis (CTAP).

There was no statistically significant relationship between presence of an imaging pathway for suspected IBD and exclusive use of non-ionising modalities in the outpatient setting (*p* = 1.00), nor for inpatient imaging within working hours (*p* = 0.54).

### Imaging in different settings

#### Acute inpatient imaging

**Figure 4.**
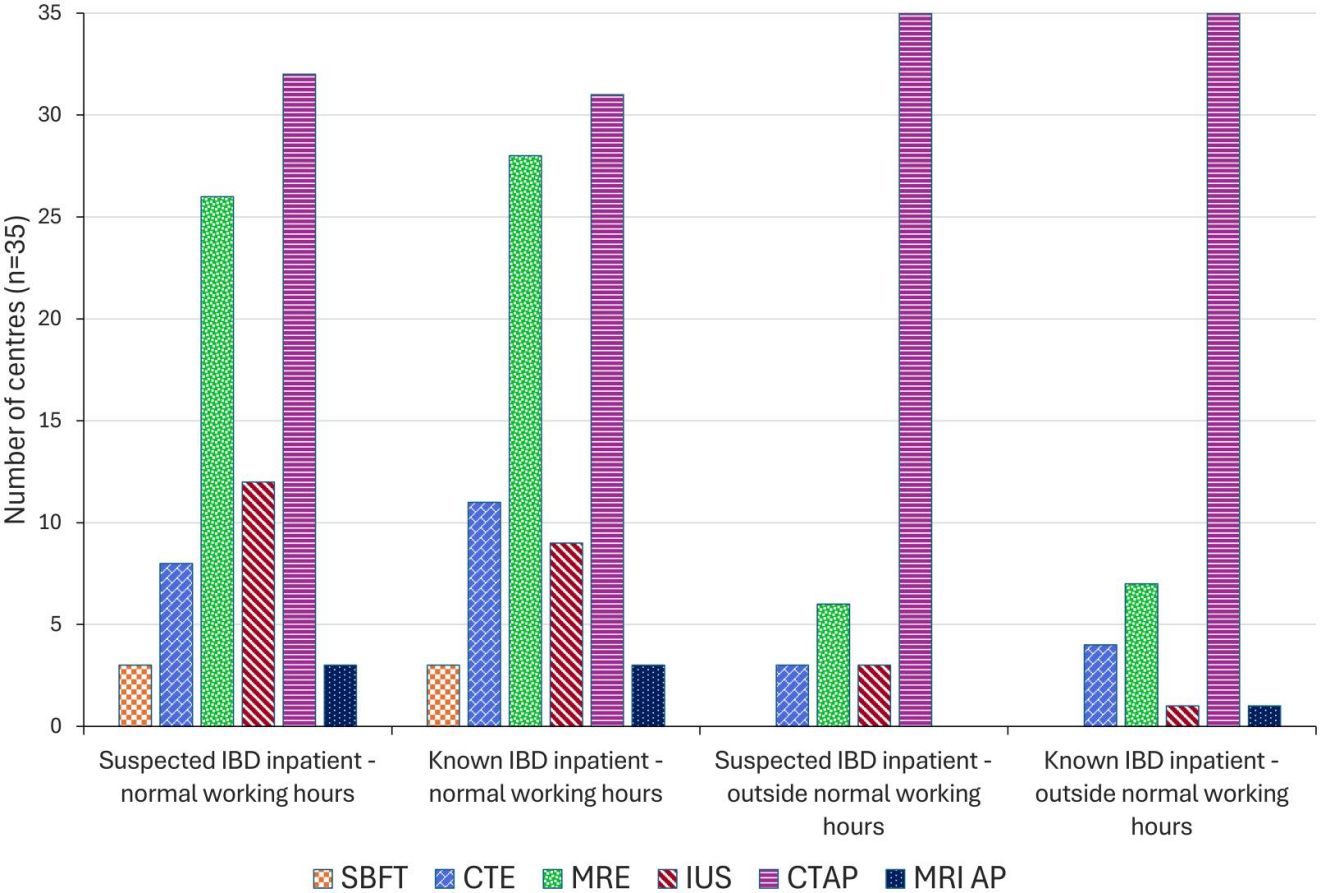
Availability and usage of different imaging modalities in patients with inflammatory bowel disease in the inpatient setting. *MRI abdomen and pelvis (MRI AP)*.

In the acute inpatient setting, CTAP was available and used by the most centres for imaging IBD both in and out-of-hours. In-hours, CTAP was used by 32/35 (91%) centres for imaging suspected IBD and by 31/35 (89%) centres for known IBD (Figure 4). This was despite centres reporting that at least one non-ionising modality (MRE or US) was also available in 27/35 (77%) centres for suspected IBD and 28/35 (80%) for known IBD.

Out-of-hours, CTAP was used by 35/35 (100%) centres for imaging both suspected and known IBD. For both suspected and known IBD, only 8/35 (23%) centres had availability of a non-ionising modality out-of-hours. The difference between availability of a non-ionising modality in-hours vs out-of-hours was statistically significant (*p* < 0.0001) both for suspected and known IBD.

##### Imaging in pregnancy

Most centres used non-ionising modalities for imaging pregnant patients with suspected and known IBD. Thirty-three of the 35 (94%) centres that responded to questions regarding imaging in pregnancy used MRE and 15/35 (43%) IUS. One centre reported using both CTE and SBFT in pregnant patients.

#### Outpatient imaging

##### Suspected IBD

**Figure 5.**
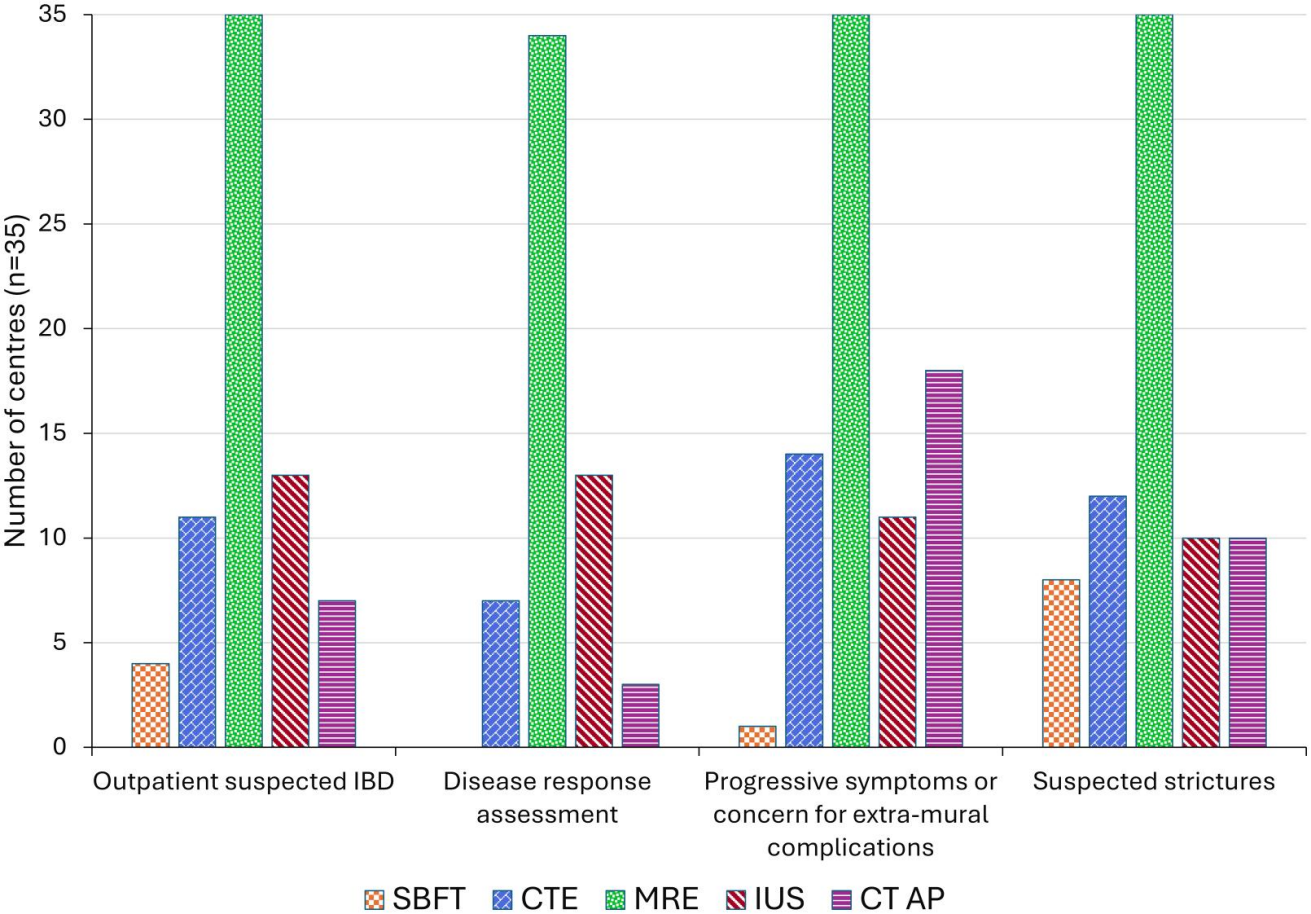
Availability and usage of different imaging modalities in patients with inflammatory bowel disease in the non-acute setting.

In outpatients, MRE was used at all centres for investigation of suspected IBD, with IUS used in 13/35 (37%) centres (Figure 5). Ionising modalities were used less frequently; CTE in 11/35 (31%), CTAP in 7/35 (20%), and SBFT in 4/35 (11%) centres.

Sixteen of the 35 centres (46%) reported exclusive use of non-ionising modalities for investigation of suspected IBD in outpatients. However, there was a statistically significant difference between use of a non-ionising imaging modality for investigation of suspected IBD in outpatients vs inpatients both in-hours (*p* = 0.005) and out-of-hours (*p* < 0.001) suggesting increased use of modalities imparting ionising radiation for inpatients.

##### Imaging at diagnosis

Cross-sectional imaging is recommended for all patients with Crohn’s disease at the time of diagnosis,^5^ and this standard was met at 29/35 (83%) centres. There was no statistically significant association between imaging always being performed at the time of diagnosis in centres with a formal imaging pathway vs those without a pathway (*p* = 0.65). There was also no statistically significant relationship between presence of an imaging pathway and use of non-ionising modalities for initial assessment (*p* = 0.47).

#### Follow-up imaging

Cross-sectional imaging was performed for assessment of disease response in asymptomatic patients with IBD at 34/35 (97%) centres. Non-ionising modalities (MRE and IUS) were used at 34/35 (97%) and 13/35 (37%) centres, respectively (Figure 5).

All centres reported using non-ionising cross-sectional imaging with MRE for patients with progressive symptoms or concern for extra-mural complications and suspected strictures. CT abdomen and pelvis was used by 18/35 (51%) centres for progressive symptoms/concern for extra-mural complications, CTE by 14/35 (40%), and IUS by 11/35 (31%). For suspected strictures, CTE was used at 12/35 (35%) centres, with IUS and CTAP both used at 10/35 (29%). All centres reported the routine use of cross-sectional imaging to assess disease extent prior to surgical resection.

#### Biologic therapies

A formal imaging pathway for the follow-up of patients on biologic therapies was present at 16/35 (46%) centres. There was no statistically significant relationship between the presence of an imaging pathway for biologics in secondary vs tertiary centres (*p* = 0.87) nor for exclusive use of non-ionising radiation imparting imaging modalities for reassessment in this patient group (*p* = 0.53). Thirty-four of the 35 (97%) centres attained the recommendation of performing a chest radiograph to screen for tuberculosis prior to commencing anti-TNF therapy. Thirty-three (94%) centres had a register for patients on biologic therapies.

Patients prescribed biologic therapies were followed up using multiple methods, including imaging in 31/35 (89%) centres, endoscopy in 30/35 (86%), and clinical assessment in 34/35 (97%). No association was demonstrated between imaging follow-up of patients prescribed biologic therapies in centres with formal imaging pathways. In the 31 centres using imaging follow-up, non-ionising modalities were reported with the highest frequency: MRE (31/31, 100%) and IUS (12/31, 39%) vs CTE (7/31, 23%), CTAP (4/31, 13%), and SBFT (0/31, 0%).

It is recommended that patients using biologic therapies have their disease reassessed at least every 12 months; however, in centres reporting the use of imaging for follow-up of these patients, there was inconsistency in the intended mean interval between repeat imaging (Table 5).^13^ There was a trend between the presence of an imaging pathway for follow-up of patients on biologic therapies and intended mean imaging interval of ≤12 months; however, this was not statistically significant (*p* = 0.07).

**Table 5.** The intended mean interval between repeat imaging in centres using imaging for follow-up of disease activity in patients taking biologic therapies.

Intended mean imaging interval	Number of centres (*n* = 31)
3-6 months	1 (3%)
6-12 months	12 (39%)
>12 months	1 (3%)
Variable	17 (55%)

## Discussion

This large national multi-centre audit has demonstrated significant variability in IBD imaging practice across radiology departments in the UK. These important findings illustrate inequality in patient access to services, highlighting the need for more imaging resources not imparting ionising radiation and further education and training within the radiology community.

Current guidelines place emphasis on the use of non-ionising imaging modalities where possible for the investigation of IBD.^4^^,^^7^ In accordance with this, MRE contributed the greatest number of total examinations performed across the UK (69.7%); however, it was frequently supplemented with other modalities, with considerable centre-by-centre variation in usage. Intestinal ultrasound is an effective imaging modality in IBD,^17^ and whilst IUS represented 17.5% of total UK examinations, it was only available at 46% of centres, with 5 high-volume centres contributing more than 80% of the IUS numbers. A few centres performed a high proportion of SBFT and CTE relative to non-ionising techniques. Whilst CTE has been demonstrated as an effective imaging modality,^7^^,^^17^ routine use of SBFT is no longer recommended.^7^^,^^11^ Of the modalities, SBFT had the lowest number of annual investigations across all centres; however, 3 centres performed 65% of all reported SBFT examinations, and SBFT comprised an average of 46% of the total examinations at those centres. This may reflect a reluctance within some centres to transition away from SBFT, historically a mainstay of IBD imaging,^6^ to recommended non-ionising modalities.

There were significant differences in imaging modalities used between inpatients and outpatients, and in- and out-of-hours. Whilst MRE use predominated in the outpatient setting, there was increased use of CTAP to investigate suspected IBD in inpatients both in-hours (*p* = 0.005) and out-of-hours (*p* < 0.0001). These results are congruent with the increased use of CT in the emergency setting for a wide range of acute conditions.^18^^,^^19^ The benefits of using CT in acutely unwell patients are addressed in ECCO-ESGAR guidelines,^7^ and include high accuracy in detecting complications, widespread availability, and shorter report turnaround time, which allow timely diagnosis to guide emergent medical or surgical management. However, the greater use of ionising radiation in inpatients is concerning, particularly in younger patient populations with IBD, who are already at higher risk of developing GI tract malignancy secondary to chronic inflammation and use of immunosuppressive therapies.^20^ We have demonstrated a statistically significant difference between the availability of non-ionising imaging modalities in-hours vs out-of-hours (*p* < 0.0001), revealing lack of access to MRI and IUS outside of normal working hours. Access to specialist radiology services is well documented, with a national shortage of radiology workforce and scanner capacity well known.^21^

This audit demonstrated variation in the type, concentration, and volume of oral contrast used for cross-sectional imaging in IBD (data in the Supplementary Material). Current guidelines do not specify a preferred oral contrast agent, although they recommend an ingested volume of 1-1.5 L.^12^ The observed variation in oral preparation across centres highlights the need for further research into the optimal contrast agent, with consideration given to the tolerability and efficacy of each agent. This may lead to more uniform image quality, improve diagnostic accuracy, and allow the application of standardised reporting indices.^5^^,^^16^

Variation in the MRE protocols used was also evident between centres. The majority included the ESGAR/ESPR recommended sequences, including post-contrast imaging, in their MRE protocols.^12^ Sixty-eight percent of centres had a dedicated non-contrast MRE protocol, although its application to different clinical scenarios was variable. Centres with established imaging pathways for known IBD were more likely to use non-contrast MRE protocols for routine follow-up. The decision of some centres to adopt a non-contrast follow-up protocol may be partially influenced by evidence linking intracranial gadolinium deposition following contrast-enhanced MRI.^2^^,^^22^ Whilst post-gadolinium sequences are currently recommended by ESGAR/ESPR and considered safe,^12^ the evidence concerning cerebral deposition presents a challenge to repeated use of post-gadolinium sequences, particularly given the younger population and more extensive imaging follow-up. Diffusion-weighted imaging has demonstrated accuracy in the detection of active inflammation in IBD and can enable diagnosis in the absence of intravenous contrast.^23^^,^^24^ Diffusion-weighted imaging was used at 73% centres and in 36% of centres with a non-contrast protocol. The susceptibility of DWI to artefact and false-positive readings may explain its variable use across UK centres.^1^ Novel MRE parameters, such as dynamic contrast-enhanced MRE and motility mapping, were not widely included in MRE protocols. However, these emerging techniques allow for quantitative assessment of disease activity and, along with IUS, may have a future role in the dynamic assessment of IBD.^2^^,^^25^

Overall, 37% centres performed MRE examinations in ≤30 minutes. There was a wide range (5-15) in the number of MRE sequences used, many deemed optional or not recommended. Whilst not currently part of formal guidelines, recent evidence suggests that simplified MRE protocols with fewer sequences do not detract from diagnostic accuracy.^26^ The addition of DWI and post-contrast sequences did not significantly improve diagnostic accuracy when compared with fat-saturated and non-fat-saturated T2w and SSFP GE sequences alone, which are useful for assessment of mural oedema in active disease. The shortened protocol conferred the additional benefit of reducing scanning and interpretation time, which, in turn, likely improves compliance and efficiency, reduces cost, and gadolinium use. There is some evidence to suggest that gadolinium administration improves the characterisation of penetrating disease.^23^^,^^26^ Therefore, the bespoke reservation of intravenous contrast administration for patients with clinical suspicion of extra-mural complications may reduce scan time for most patients who have uncomplicated disease.

Imaging plays a crucial role in the long-term follow-up of patients with IBD.^2^ There is currently a lack of evidence and formal guidance on the optimal method and interval of follow-up, particularly for patients prescribed biologic therapies. This is reflected in our results, which demonstrated that whilst 46% of centres had a formal imaging pathway for follow-up of patients on biologic treatments, there was variability in the parameters, modalities, and timing of follow-up. The more widespread use of biologic treatments mandates further research and consensus agreement on a standardised follow-up pathway for this patient group.

The results of this audit show there is significant scope for improvement in IBD imaging. Locally agreed diagnostic pathways have been proposed to streamline investigation. Although the number and range of modalities used to investigate suspected IBD were broadly similar in centres with and without established pathways, this audit was not designed to test the effectiveness of such pathways. We have demonstrated that whilst MRE was the most widely used modality, it also had the lowest number of centres achieving the IBD UK target 4-week waiting time (38%) and had the highest median wait time across all modalities.^4^ The significant time and cost implications of MRI, combined with limitations in MRI scanner capacity, may preclude major upscaling of MRE without significant investment in infrastructure. Equally, ongoing training and education is important for radiologists, as the number of reporters ranged widely, up to 19 in one centre.

More widespread access to IUS is one proposed avenue for improvement and may be more attainable than increasing MRI capacity. Of all the modalities investigated, IUS was available at the fewest centres (46%) but may be an overestimate because of selection bias of sites contributing to the audit. There was substantial variability in the training received by IUS practitioners. Improved access to IUS training and the development of standardised IUS training accreditation could enable the more widespread incorporation of IUS into formalised IBD imaging pathways, thus improving patient access.

This retrospective audit has some limitations. Missing data from centres limited the statistical analyses that were performed, particularly in relation to pathways after biological treatment. The design of the second section of the questionnaire requested centres to list all modalities used in different settings (inpatient vs outpatient), at different times of day (in-hours and out-of-hours) and in different patient groups (suspected but undiagnosed IBD, known IBD, follow-up, etc) as opposed to the total number of examinations performed. It was, therefore, not possible to assess the overall reliance of different centres on each modality in different clinical settings. Such data would be challenging for individual centres to collect and require extensive scrutinisation of imaging request forms and clinical notes.

In conclusion, this national multi-centre audit has highlighted substantial variation in IBD imaging practice across the UK. There remains considerable reliance on ionising imaging in inpatients and generalised lack of access to non-ionising modalities out-of-hours. Further research to optimise imaging pathways for greater uniformity in care is needed, and improved resources for education and training should be prioritised.

## Supplementary Material

tqaf050_Supplementary_Data
