# Supplementary material for: A British Society of Gastrointestinal and Abdominal Radiology multi-centre audit of imaging investigations in inflammatory bowel disease
Source: Br J Radiol. 2025 Mar 10;98(1169):734–43. doi: 10.1093/bjr/tqaf050 (PMC12012338; doi:10.1093/bjr/tqaf050)
Supplement: tqaf050_Supplementary_Data [file tqaf050_supplementary_data.zip › tqaf050_Supplementary_Data/Supplementary Material_revision.pdf]

Supplementary Methods

Table of audit standards

Table 1. Table of audit standards. *Abbreviations used: multidisciplinary team (MDT), inflammatory bowel disease (IBD), magnetic resonance imaging (MRI), computed tomography (CT), intestinal ultrasound (IUS), magnetic resonance enterography (MRE), computed tomography enterography (CTE), intravenous (IV), TNF (tumour necrosis factor alpha).*

	**Standard**
**The MDT**	
1	Patients should be cared for by a defined IBD multidisciplinary team led by a named consultant adult or paediatric gastroenterologist.[1]
2	The IBD multidisciplinary MDT should include a core membership consisting of gastroenterologist, colorectal surgeon, IBD specialist nurse, radiologist, dietitian, histopathologist and pharmacist, all of whom should have expertise in IBD.[2]
3	Multidisciplinary team meetings should take place regularly to discuss appropriate patients.[1]
4	For each IBD patient discussed in the MDT meeting, a formal record should be kept of management decisions reached.[2]
**Choice of imaging modality**	
5	Cross-sectional imaging, specifically MRI, CT and ultrasound, have largely replaced conventional barium fluoroscopic and nuclear medicine techniques and have the advantage of evaluating both luminal and extraluminal disease. Emphasis should be placed on MRI enterography and ultrasound as they do not expose patients to ionising radiation.[2]
6	IUS and abdominal MRI without intravenous gadolinium are the safest techniques to examine pregnant women in whom IBD is known or suspected, regardless of the trimester.[3]
**Patient preparation for MRE and CTE**	
7	It is recommended that patients should not eat any solid food for 4-6 hours.[4]
8	There is no single preferred contrast agent for MRE or CTE. Recommended agents include mannitol (with or without locust bean gum), polyethylene glycol, sorbitol and lactulose amongst others.[4]
9	The optimal volume of oral contrast is 1,000-1,500 ml.[4]
10	It is recommended that ingestion time of oral contrast without previous major small bowel resection should be 46-60 minutes.[4]
**MRE technical protocols**	
11	Spasmolytic agents:<br>• It is recommended that spasmolytic agents are administered during MRE.[4]<br>• The recommended first line spasmolytic agent is IV hyoscine butylbromide.[4]<br>• The recommended dose of IV hyoscine butylbromide is 20 mg.[4]
12	The following MRE sequences are recommended[4]:<br>• Axial and coronal fast spin echo (FSE) T2-weighted sequences without fat saturation.<br>• Axial and coronal steady state free precession gradient echo (SSFP GE) sequences without fat saturation.<br>• An axial or coronal FSE T2-weighted sequence with fat saturation.<br>• Non-enhanced coronal T1-weighted sequence with fat saturation followed by contrast-enhanced coronal and axial T1-weighted sequences with fat saturation.<br>• In patients with known or suspected inflammatory bowel disease, contrast-enhanced sequences should be in the enteric (45 seconds) or portal venous phase (70 seconds).
13	The following sequences are optional[4]:<br>• An additional FSE T2-weighted sequence with fat saturation.<br>• Axial and coronal SSFP GE sequences with fat saturation.<br>• Cine motility.

	• Diffusion weighted imaging.
14	It is recommended that diffusion-weighted sequences should include lower b values ranging from 0 or 50 and upper b values ranging from 600 to 900.[4]
15	Coronal diffusion-weighted sequences are not recommended.[4]
16	If cross-sectional enterography is indicated and intravenous contrast cannot be administered, non-contrast MRE with T2-weighted and diffusion-weighted imaging should be used an acceptable alternative.[5]
17	It is recommended that in general the total acquisition time for should be equal to or less than 30minutes.[4]
**CTE technical protocols**	
18	Multidetector CT with at least 64 slices is optimal.[4]
19	Spasmolytic agents[4]:<br>• The use of a spasmolytic agent during CTE is optional.<br>• It is recommended that if a spasmolytic is used the first line agent is IV hyoscine butylbromide.<br>• The recommended dose of IV hyoscine butylbromide is 20mg.
20	It is recommended that the maximal slice thickness for displaying axial, coronal and sagittal reconstructed images should be 3mm.[4]
21	Intravenous contrast[4]:<br>• It is recommended that either an enteric phase or portal venous phase acquisition is performed.<br>• Additional acquisitions including pre-contrast, arterial, and delayed phase (6-7minutes) are not recommended.<br>• It is recommended that the IV iodinated contrast iodine dose should be varied according to the patients' weight at 1.5ml/kg.
**IUS technical protocols**	
22	It is recommended that patients should be nil by mouth for solids for 4-6 hours.[4]
23	It is recommended that evaluation with both low and high frequency probes is performed.[4]
24	Hydrosonography preparation[4]:<br>• It is not recommended that laxative bowel preparation is administered.<br>• The use of a spasmolytic agent is not recommended.<br>• There is no single preferred contrast agent for hydosonography. Recommended agents include mannitol (with or without locust bean gum), polyethylene glycol, sorbitol and lactulose amongst others.<br>• It is recommended that the optimal volume of oral contrast for should exceed 500ml.<br>• It is recommended that ingestion time of oral contrast should be 45 minutes.
25	It is recommended to routinely use colour Doppler.[4]
26	Routine use of IV IUS contrast agent is not recommended.[4]
27	Dedicated training in bowel ultrasound is necessary and should be performed following training in general abdominal ultrasound.[6]
**Imaging in known IBD**	
28	New diagnosis:<br>• All newly diagnosed Crohn's disease patients should undergo small bowel assessment (MRE, IUS and/or capsule endoscopy).[4]<br>• Cross-sectional enterography should be performed at diagnosis of Crohn's disease to detect small bowel inflammation and penetrating complications beyond the reach of standard ileocolonoscopy.[5]
29	Endoscopic assessment and ultrasound/MRI/CT/contrast studies should be accessible within 4 weeks, and within 24 hours where patients are acutely unwell or require admission to the hospital.[1]
30	Endoscopic or cross-sectional reassessment in Crohn's disease should be considered in cases of relapse, persistent disease activity, new unexplained symptoms, and prior to switch of therapy.[3]
**Imaging IBD on treatment**	
31	Prior to commencing anti-TNF therapy, IBD patients should be screened for tuberculosis (TB) using a combination of clinical risk stratification, chest x-ray and interferon-gamma release assays.[2]
32	Cross-sectional enterography should be considered in disease monitoring paradigms when small bowel disease or penetrating disease complications are present.[5]

33	MRE should be used rather than CTE, when possible, for estimating response to medical treatment in asymptomatic Crohn's disease, as its multiparametric nature permits evaluation of multiple imaging parameters that reflect inflammation and avoids radiation.[5]
34	Monitoring patients on biologic treatment[7]:<br>• Treatment with infliximab or adalimumab should only be continued if there is clear evidence of ongoing active disease as determined by clinical symptoms, biological markers and investigation, including endoscopy if necessary.<br>• People who continue treatment with infliximab or adalimumab should have their disease reassessed at least every 12 months to determine whether ongoing treatment is still clinically appropriate.
35	Patients with IBD should have assessment and optimisation of their physical condition prior to elective surgery. This should include appropriate imaging to determine disease extent and complications.[2]
**Imaging for complications**	
36	Extramural complications in Crohn's disease (such as fistulae and abscesses) should be monitored by cross-sectional imaging, including IUS or MRI (or both) in combination with clinical and laboratory parameters.[3]
37	Cross-sectional imaging should be used to detect small bowel strictures. Due to radiation exposure with CT, the preferred methods are MRI and/or IUS.[3]
**Reporting**	
38	Radiologists interpretating cross-sectional imaging in IBD require appropriate training with initial evidence suggesting the radiologist should review at least 100 cases.[6]
39	Reporting of findings should be structured to improve communication to clinicians, ensure inclusion of all important disease features, and improve report structure and reproducibility.[8]
40	Reporting of imaging parameters[7]:<br>• The number and anatomical location of intestinal segments with imaging findings of mural inflammation should be reported, including skip lesions. An estimate of the total affected length and length of all individual pathological areas of the small bowel is preferred.<br>• Thickness of the most involved small bowel and/or colonic segment, defined as bowel wall thickness (BWT), should be measured and reported. A threshold of 3 mm is the recommended cut-off for presence of mural inflammation for both small and large bowel.

Supplementary Results

Magnetic resonance enterography (MRE)

Patient preparation

Thirty-two centres provided data on patient fasting prior to MRE, of which 30/32 (94%) centres advised fasting for the recommended 4-6 hours. One centre did not suggest fasting, and one advised a 12-hour fasting period.

Mannitol was the most common oral preparation agent, used by 24 of 33 (73%) responding centres, with one centre using both mannitol and polyethylene glycol. There was considerable variation in the other oral preparation agents used and in the concentration of preparation used (Table 2). The recommended optimal volume of oral contrast ingestion of 1-1.5L was protocolled in 15/33 (45%) centres. Oral preparation was protocolled to be ingested 45-60 minutes prior to scanning as per the standard in 21/33 (64%) centres.

All 36 responding centres used hyoscine butylbromide as the first line anti-spasmolytic, with 31/36 (86%) using the recommended dose of 20mg. Three centres used 10mg and two centres used 40mg split into two 20mg doses.

Table 2. Oral contrast agents and concentrations protocolled for MR enterography.

		**Concentration**			
**Oral preparation**	**Number of centres using agent (n=33)**	**<1.5%**	**2%**	**2.50%**	**>3%**
Mannitol	24	5	2	10	7
Polyethylene glycol (PEG)	4	1			3
Lactulose	3	1			2
Klean prep	3		2	1	

Computed tomography enterography (CTE)

Patient preparation

Eighteen centres (44%) provided complete data on patient preparation for CTE. Mannitol was the most used oral preparation agent, and the most common concentration used was >3% (Table 3). One centre used two different agents: mannitol and Klean prep. The recommended 1-1.5L of oral preparation ingestion was protocolled in 9/18 (50%) centres.

Table 3. Oral contrast agents and concentrations protocolled for CT enterography.

		**Concentration**			
**Oral preparation concentration**	**Number of centres (n=18)**	**<1.5%**	**2%**	**2.50%**	**>3%**
Mannitol	15	1	2	5	7
Polyethylene glycol (PEG)	2	1			1
Klean prep	1			1	
Lactulose	1	1			

Intestinal ultrasound (IUS)

Patient preparation

Of the 20 centres that reported using IUS for investigating intestinal ultrasound (IBD), 17 centres provided further information regarding their IUS protocol. Routine patient fasting for 4-6hrs prior to IUS was advised by 9/17 (53%) centres. Two centres routinely gave oral contrast, using Klean prep and water respectively, both at a volume of 500-1000ml. No centres reported the use of spasmolytic agents, rectal enemas, laxatives or intravenous contrast.

Multi-disciplinary team (MDT) working

Thirty-nine centres provided information regarding their local IBD MDT, which occurred on a weekly basis at 22/39 centres (56%) (Table 4). Radiologists were present at all MDTs in 35/39 (90%) centres and 34/39 (87%) have the allocated time for MDT activity in their job plan. MDT outcomes were formally recorded at 34/39 centres (87%).

Table 4. Frequency of IBD MDT across secondary and tertiary centres (n=39).

	**Weekly**	**Fortnightly**	**Monthly**	**>Monthly**
Secondary care centres	9 (23.1%)	5 (12.8%)	7 (17.9%)	1 (2.6%)
Tertiary care centres	13 (33.3%)	3 (7.7%)	1 (2.6%)	0
Total	22 (564%)	8 (20.5%)	8 (20.5%)	1 (2.6%)

Supplementary Materials References

1. Kapasi R, Glatter J, Lamb CA, et al. Consensus standards of healthcare for adults and children with inflammatory bowel disease in the UK. *Frontline Gastroenterol*. 2020;11(3):178-187. doi:10.1136/flgastro-2019-101260

2. Lamb CA, Kennedy NA, Raine T, et al. British Society of Gastroenterology consensus guidelines on the management of inflammatory bowel disease in adults. *Gut*. 2019;68:s1-s106. doi:10.1136/gutjnl-2019-318484

3. Maaser C, Sturm A, Vavricka SR, et al. ECCO-ESGAR Guideline for Diagnostic Assessment in IBD Part 1: Initial diagnosis, monitoring of known IBD, detection of complications. *J Crohns Colitis*. 2019;13(2):144-164. doi:10.1093/ecco-jcc/jjy113

4. Taylor SA, Avni F, Cronin CG, et al. The first joint ESGAR/ ESPR consensus statement on the technical performance of cross-sectional small bowel and colonic imaging. *Eur Radiol*. 2017;27(6):2570-2582. doi:10.1007/s00330-016-4615-9

5. Bruining DH, Zimmermann EM, Loftus E V., et al. Consensus recommendations for evaluation, interpretation, and utilization of computed tomography and magnetic resonance enterography in patients with small bowel Crohn's disease. *Radiology*. 2018;286(3):776-799. doi:10.1148/radiol.2018171737

6. Sturm A, Maaser C, Calabrese E, et al. Ecco-esgar guideline for diagnostic assessment in ibd part 2: Ibd scores and general principles and technical aspects. *J Crohns Colitis*. 2019;13(3):273-284E. doi:10.1093/ecco-jcc/jjy114

7. *Crohn's Disease: Management NICE Guideline*.; 2019. www.nice.org.uk/guidance/ng129

8. Kucharzik T, Tielbeek J, Carter D, et al. ECCO-ESGAR Topical Review on Optimizing Reporting for Cross-Sectional Imaging in Inflammatory Bowel Disease. *J Crohns Colitis*. 2022;16(4):523-543. doi:10.1093/ecco-jcc/jjab180
